# Supplementary figures and images for: Didemnin B and ternatin-4 differentially inhibit conformational changes in eEF1A required for aminoacyl-tRNA accommodation into mammalian ribosomes
Source: eLife. 2022 Oct 20;11:e81608. doi: 10.7554/eLife.81608 (PMC9584604; doi:10.7554/eLife.81608)

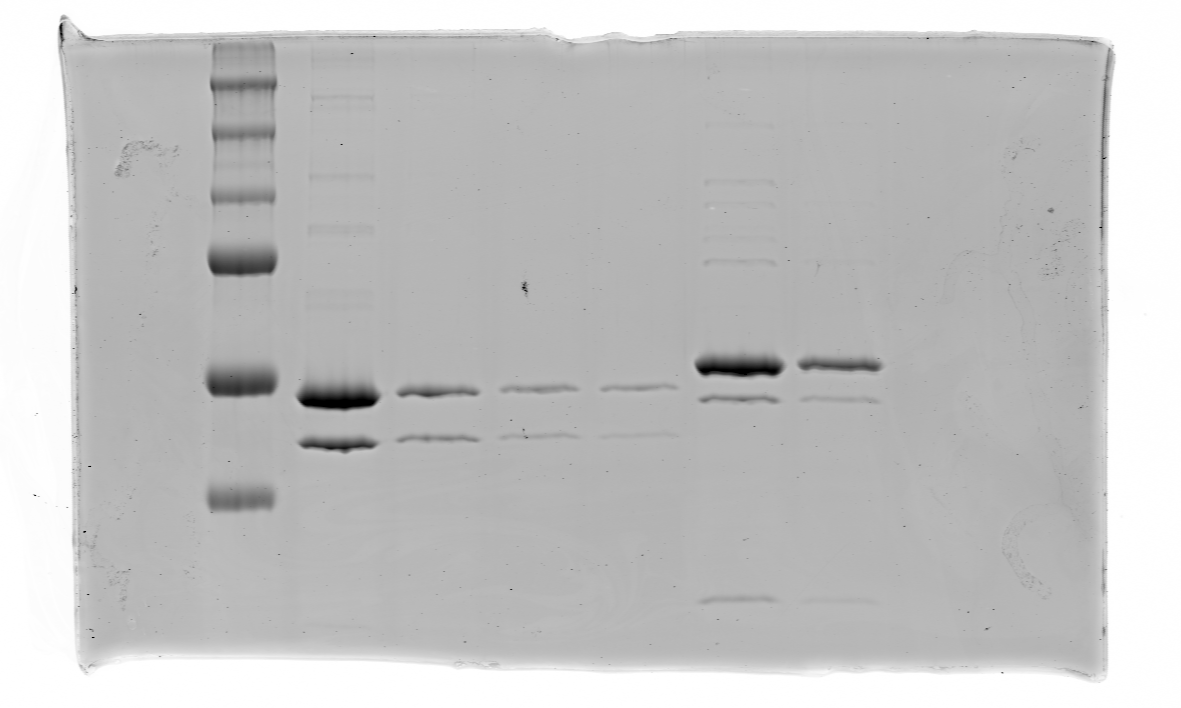

Supplement: Figure 1—figure supplement 3—source data 1. [file elife-81608-fig1-figsupp3-data1.zip › Figure1_supplement3A_gel/EF1A_purification_raw_700.tif]
